# Reply To: Confined molecular catalysts provide an alternative interpretation to the electrochemically reversible demetallation of copper complexes

**DOI:** 10.1038/s41467-022-31662-0

**Published:** 2022-07-22

**Authors:** Zhe Weng, Yueshen Wu, Maoyu Wang, Gary W. Brudvig, Victor S. Batista, Yongye Liang, Zhenxing Feng, Hailiang Wang

**Affiliations:** 1 Department of Chemistry and Energy Sciences Institute, Yale University, New Haven, Connecticut 06511 USA; 2 School of Chemical, Biological, and Environmental Engineering, Oregon State University, Corvallis, OR 97331 USA; 3 Department of Materials Science and Engineering, South University of Science and Technology of China, Shenzhen, 518055 China; 4 Present Address: Tianjin University, Tianjin, China; 5 Present Address: Twelve, Berkeley, CA USA; 6 Present Address: Argonne National Laboratory, Lemont, IL USA

**Keywords:** Electrocatalysis, Electrocatalysis, Nanoscale materials

**replying to** E. Boutin et al. *Nature Communications* 10.1038/s41467-022-31661-1 (2022)

In the Matters Arising (MA) by Robert and Boutin (henceforth abbreviated RB) on our article entitled “Active Sites of Copper-Complex Catalytic Materials for Electrochemical Carbon Dioxide Reduction” published in *Nature Communications* more than four years ago in January 2018 (the article)^1^, RB questioned our interpretation of a portion of our results and offered an alternative explanation. Specifically, based on our in situ X-ray absorption spectroscopy (XAS) results, we proposed that at reductive electrode potential (−1.06 V vs RHE, all potentials below are with respect to RHE unless otherwise stated), reduction of copper phthalocyanine (CuPc) leads to the formation of small Cu nanoparticles (NPs) that are electrocatalytically active for CO_2_ reduction to CH_4_; subsequently, after release of the reductive potential, oxidation of the Cu NPs and re-coordination with the empty Pc ligands occur to regenerate CuPc (Table 1). It is this latter point that RB disagree with. They argue that upon reoxidation, all the Cu NPs leach into the electrolyte as Cu^2+^ ions and the XAS signal for CuPc arises solely from the unreacted part of CuPc that is not reduced at the previous reductive potential (Table 1). However, in their MA, RB do not provide any original data; nor do they show any evidence that can directly support their argument. Therefore, the original results and conclusions of the article are not affected. Nevertheless, we would like to take this opportunity to update readers with additional data we collected in our earlier experiments as well as results from more recently published independent studies from other research groups that have directly confirmed our conclusions in the article.

**Table 1:** Original conclusions of the article and key points raised by RB’s MA.

Our original conclusions	RB’s MA
At reductive potential (−1.06 V), CuPc is reduced to Cu NPs which are the active catalyst for CO_2_ reduction to CH_4_.	Agrees with us on what happens at the reductive potential.
*After release of the reductive potential, (at least a large part of) Cu NPs are converted back to CuPc*.	*After release of the reductive potential, All Cu NPs are oxidized to Cu*^*2+*^ *and leach into the electrolyte, no regeneration of CuPc*.

Figure. 1 shows the original unnormalized XAS spectra of our CuPc electrode at the initial open circuit voltage (OCV), at −1.06 V and then back to OCV. The unnormalized edge jumps of the Cu K absorption spectra can be used to quantitatively compare the amount of Cu species on the electrode. At the initial OCV, the unnormalized intensity is 1.5 with 100% of the Cu element in the CuPc form, corresponding to the initial state. At −1.06 V, the absorption intensity has decreased to 1.2, which is not uncommon for this kind of multi-hour measurement^2,3^. One likely reason for the loss is gas bubbling stripping active material off the electrode. Analysis shows that the material at −1.06 V contains 80% Cu NPs and 20% CuPc, which directly leads to the conclusion that a large part of the CuPc on the electrode has been reduced to Cu NPs. After measurements at negative potentials, the electrode was left at OCV (labeled as OCV-2 to distinguish from the initial OCV) for several hours before the final XAS spectrum was taken. Note that the 0.64 V label in the original article was inaccurate and the description “upon release of the negative electrode potential” was ambiguous (0.64 V was the value of the initial OCV which should differ from that of OCV-2; we apologize to the readers for this negligence). At OCV-2, the absorption intensity has further decreased to 0.8 with 100% CuPc. Note that at −1.06 V the unreacted CuPc accounts for an intensity of 0.24 (20% of total intensity 1.2). Therefore, at least a significant part of the Cu NPs at −1.06 V has been converted back to CuPc (conclusion of the article). It is impossible that the CuPc signal at OCV-2 is entirely from unreacted CuPc (the RB claim) whose theoretical maximum contribution to absorption intensity is 0.24. These experimental results directly prove that RB’s argument in their MA is incorrect.

**Fig. 1:**
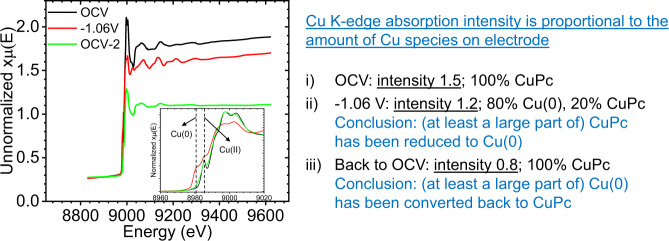
Unnormalized in-situ XAS spectra. CuPc under electrochemical CO_2_ reduction conditions with the near edge structure shown as inset (same original data that generated Fig. 2 in the article).

Over the past several years since the publication of the article, there have been a considerable number of independent studies from other research groups that directly or indirectly confirm our restructuring conclusions. In their work published in *ChemSusChem* in 2020, Mougel and Fontecave et al. studied a polymer of CuPc coated on carbon nanotubes for electrochemical CO_2_ reduction^4^. With in-situ XAS, they also observed reduction to Cu NPs under working conditions and restoration of the original CuN_4_ coordination structure upon reoxidation (Fig. 2a, b). In another work by Min, Lin, Zhu and co-workers published in *Nature Communications* in 2021, the authors performed in-situ UV-vis spectroscopic measurements of CuPc under electrochemical CO_2_ reduction reaction conditions^5^. At reducing electrode potentials < −1.04 V, the CuPc absorption features almost disappear and the B-bands of free-base Pc dominate (Fig. 2c). After release of the reduction potential, the UV-vis profile returns to that of CuPc, which directly confirms our reversible restructuring finding with a different technique. Similar reversible restructuring between single Cu(II) sites and Cu(0) NPs have also been observed for CO_2_ reduction electrocatalysts that can be considered analogs of CuPc, such as single CuN_4_ sites embedded in a carbon network^6^ and CuN_2_Cl_2_ in a covalent triazine framework^7^. More studies as such are summarized in a recent short review article published in *Nature Communications*^8^. All these independent studies clearly support our conclusions in the article and testify directly against RB’s argument. Unfortunately, the results and conclusions of these important studies were overlooked, intentionally or otherwise, by RB.

**Fig. 2:**
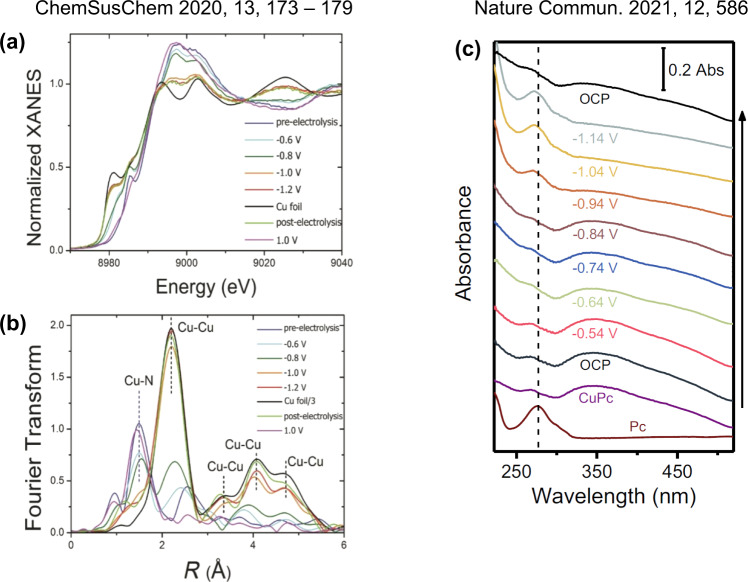
Two representative recent studies from other research groups that directly support our conclusions. **a**, **b** In-situ XAS results (**a** near edge absorption; **b** Fourier transform of the extended range) of a CuPc polymer catalyst under electrochemical CO_2_ reduction conditions. Adapted with permission from Ref. ^4^. Copyright 2020 Wiley. **c** In-situ UV-vis study of CuPc under electrochemical CO_2_ reduction conditions. Adapted with permission from Ref. ^5^.

In conclusion, the reversible restructuring postulation remains as the best interpretation of the results, which is supported by our experimental data and has been confirmed by other independent studies.

## Data Availability

The data that support the findings of this study are available within the paper or are available from the corresponding authors upon reasonable request.
